# Effects of ebselen addition on emotional processing and brain neurochemistry in depressed patients unresponsive to antidepressant medication

**DOI:** 10.1038/s41398-024-02899-8

**Published:** 2024-05-07

**Authors:** Fitri Fareez Ramli, Nisha Singh, Uzay E. Emir, Luca M. Villa, Shona Waters, Catherine J. Harmer, Philip J. Cowen, Beata R. Godlewska

**Affiliations:** 1 Psychopharmacology Research Group, Department of Psychiatry, University of Oxford, Warneford Hospital, Oxford, UK; 2 Oxford Health NHS Foundation Trust, Oxford, UK; 3 Department of Pharmacology, Faculty of Medicine, Universiti Kebangsaan Malaysia, Kuala Lumpur, Malaysia; 4 Department of Paediatrics, University of Oxford, Oxford, UK; 5 School of Health Sciences, College of Health and Human Sciences, Purdue University, West Lafayette, IN USA; 6 QYNAPSE SAS, 2-10 Rue d’Oradour-sur-Glane, Paris, France

**Keywords:** Clinical pharmacology, Predictive markers, Molecular neuroscience

## Abstract

Lithium is an effective augmenting agent for depressed patients with inadequate response to standard antidepressant therapy, but numerous adverse effects limit its use. We previously reported that a lithium-mimetic agent, ebselen, promoted a positive emotional bias—an indicator of potential antidepressant activity in healthy participants. We therefore aimed to investigate the effects of short-term ebselen treatment on emotional processing and brain neurochemistry in depressed patients with inadequate response to standard antidepressants. We conducted a double-blind, placebo-controlled 7-day experimental medicine study in 51 patients with major depressive disorder who were currently taking antidepressants but had an inadequate response to treatment. Participants received either ebselen 600 mg twice daily for seven days or identical matching placebo. An emotional testing battery, magnetic resonance spectroscopy and depression and anxiety rating scales were conducted at baseline and after seven days of treatment. Ebselen did not increase the recognition of positive facial expressions in the depressed patient group. However, ebselen increased the response bias towards fear emotion in the signal detection measurement. In the anterior cingulate cortex, ebselen significantly reduced the concentrations of inositol and Glx (glutamate+glutamine). We found no significant differences in depression and anxiety rating scales between visits. Our study did not find any positive shift in emotional bias in depressed patients with an inadequate response to antidepressant medication. We confirmed the ability of ebselen to lower inositol and Glx in the anterior cingulate cortex. These latter effects are probably mediated through inhibition of inositol monophosphatase and glutaminase respectively.

## Introduction

Major depressive disorder (MDD) is a debilitating condition affecting over 250 million people globally [1]. Selective serotonin reuptake inhibitors (SSRIs) are the recommended first line pharmacological therapy for clinical depression but produce low remission rates (about 30%) in ‘real world’ studies [2]. Switching to a second antidepressant helps a further 20% of patients achieve remission [2]. However, this leaves a substantial number of people with persistent, clinically significant symptomatology. In this situation, pharmacological ‘augmentation’ strategies are often employed where another agent is added to the ongoing antidepressant treatment.

Augmentation with lithium is one of the proposed treatment steps to help patients with difficult to treat depression achieve remission. Lithium has been reported to be an effective augmenting agent in this situation, with an additional benefit of suicide prevention [3, 4]. While lithium produces numerous neurobiological effects, one widely proposed mechanism of action is inhibition of the enzyme inositol monophosphatase (IMPase).

Based on Berridge’s inositol depletion hypothesis, lithium via IMPase inhibition reduces the inositol pool essential for producing phosphatidylinositol bisphosphate, a substrate for second messenger production [5]. Such a decrease in second messenger activity may modify the neural activity of downstream pathways. In animal models, lithium promotes behavioural and molecular changes, particularly in serotonin neurotransmission, consistent with antidepressant activity [6]. However, given lithium’s problematic side effects and the requirement of regular blood monitoring, its use in patients with depression is limited [7]. Therefore, a search was made for a pharmacological agent with a similar mechanism but potentially fewer side effects. Singh and colleagues identified the clinically safe antioxidant and IMPase inhibitor, ebselen, as a potential replacement for lithium [8].

Ebselen is a synthetic organoselenium compound, which has been reported to exert lithium-like effects in pre-clinical models including those of serotonin activity [6, 9]. In humans, using proton magnetic resonance spectroscopy (MRS), we found that a 2-day ebselen treatment lowered levels of inositol in the anterior cingulate cortex (ACC). In one of these studies, we also found a significant decrease in Glx (a combined measure of glutamate and glutamine) [10], consistent with the ability of ebselen to inhibit the enzyme, glutaminase [11]. In healthy participants, we found that ebselen treatment increased the recognition of positive emotions, replicating the positive bias shift typically observed in early antidepressant therapy [12, 13]. In addition, ebselen was well-tolerated and no serious adverse effects were reported among participants [10, 12, 13].

Given the promising effect of ebselen on positive shifts in emotional processing and brain neurochemistry and its good tolerance, we carried out an experimental medicine study in depressed patients unresponsive to standard antidepressant medication, to assess the potential of ebselen as an add-on treatment. We utilised a double-blind, placebo-controlled, experimental medicine approach to investigate the effects of a 7-day ebselen treatment regime on emotional processing and brain neurochemistry in depressed patients with inadequate response to current antidepressant therapy.

## Methods

### Participants and study design

The study was approved by the National Research Ethics Service Committee (NRES), South-Central Oxford (ID:20/SC/0151) and was registered with the US National Institutes of Health (NIH) (www.clinicaltrials.gov Identifier: NCT05117710). Informed consent was obtained from all participants before the study. All participants were screened using the Structured Clinical Interview for Diagnostic and Statistical Manual of Mental Disorders (DSM-5) (SCID-5). Fifty-one patients who met criteria for major depressive disorder and who had a score of at least 14 on the 17-item Hamilton Depression Rating Scale (HAM-D) with an inadequate response to current antidepressant treatment given at a therapeutic dose for at least four weeks were included. The sample size was calculated based on the effect size seen in previous studies of the effect of 7 days’ antidepressant treatment on the emotional face recognition task [14]. This indicated that a sample size of 25 participants receiving active treatment would provide a power of 0.9 to detect a similar effect size of around 0.4.

We excluded participants with a history of or current psychosis, bipolar disorder and emotionally unstable personality disorder, history of significant alcohol or substance dependence over the past six months, clinically significant risk of suicide, ongoing or history of electroconvulsive therapy for the current depressive episode, failure to respond to standard pharmacological augmentation treatments (lithium and atypical antipsychotics), severe claustrophobia, contraindications to magnetic resonance spectroscopy (MRI) scanning, current pregnancy and lactation, previous study participation involving emotional processing tasks or interventional medication within the last three months, and body mass index (BMI—kg/m^2^) outside the 18–36 range. Participants with medical conditions on currently prescribed medications were included if these did not compromise the safety or affect data quality. Participants were asked to maintain usual sleep and diet routines and refrain from alcohol consumption during study involvement. Premenopausal women and male participants engaging in sex with a risk of pregnancy were required to agree to use a highly effective method of contraception for 30 days after receiving the study medication treatment. Male participants agreed not to donate sperm during this time.

Ebselen and the matching placebo were provided by Sound Pharmaceuticals (Seattle, USA). Participants were randomised into ebselen and placebo groups with sequential assignment of subject numbers as the participants entered the study in a 1:1 allocation, stratified based on sex (male and female) and number of prior antidepressant treatments (1–2 vs >2). During the first study visit, participants were evaluated using an emotional testing battery, magnetic resonance spectroscopy (MRS) and depression and anxiety rating scales. These served as ‘baseline’ measures. A urine pregnancy test was conducted on female participants. Any baseline adverse effects were recorded for comparison to the second visit. Each participant received either ebselen (200 mg) or a matching placebo, and was instructed to take three capsules twice daily for seven days. The participants were re-tested after a 7-day treatment period when the same procedures were repeated. Also, at the second visit, venous blood was withdrawn for serum selenium testing, and a ‘guess treatment’ questionnaire was given. Selenium levels were measured as a surrogate ebselen level as a previous study reported a significant correlation between selenium and ebselen (and its metabolites) levels [13]. The primary outcome of the study was performance on the facial expression recognition task (see below).

### Emotional testing battery

The emotional testing battery (ETB) used in this study consists of five tasks, including a facial expression recognition task (FERT), emotional categorization task (ECAT), emotional recall task (EREC), emotional recognition memory task (EMEM) and facial dot-probe task (FDOT) [13, 14]. In the FERT task, participants were asked to indicate the emotions of each facial expression presented on the computer screen. There were six basic emotions—happy, surprise, sad, fear, disgust, and anger. Each emotion had varying intensities, from 10 to 100%, with a 10% increment. There were four examples of each emotion in each intensity, making up 40 for each emotion. Ten additional faces represented a neutral facial expression, making up to 250 presentations. The accuracy, misclassification and reaction time were recorded. We also calculated d prime (d’) and beta values as the measures for signal detection.

The ECAT task involved positive and negative valence words presented briefly on the computer screen. Before the task, participants were asked to imagine overhearing someone describing them using the presented word. The participants were instructed to press the like or dislike button as accurately and rapidly as possible when seeing the words. The accuracy and reaction time were recorded.

The third task was the FDOT to measure attentional vigilance scores in masked and unmasked conditions for happy and fearful stimuli. The task began with the presentation of a central X. Then, two faces (one neutral and one either fearful or happy) appeared on the screen, with one at the top and another at the bottom. After the faces disappeared, the two dots oriented horizontally or vertically appeared either at the top or bottom and the participants were asked to indicate the orientation by pressing the correct button. We calculated the attentional vigilance score by deducting the mean reaction times between trials when probes appeared in the same position as the emotional face (happy or fearful) and trials when probes appeared in the opposite position to the emotional face (neutral). The incorrect responses were eliminated from the analysis.

After FDOT, the participants were instructed to remember and write words from the ECAT task on the paper within four minutes. The number of correct (accuracy) and incorrect (false response) words was recorded in the EREC task. EMEM (a measure of recognition accuracy) was the last task, where participants were asked to indicate whether the words presented on the screen had been presented before (yes or no). The accuracy, misclassification and reaction time were recorded.

### Magnetic resonance spectroscopy

The Proton MRS scanning was conducted at the Oxford Centre for Human Brain Activity (OBHA) using 3T SIEMENS MAGNETOM equipped with a 32 channel receive array head coil (Siemens, Erlangen, Germany). An 8-ml voxel (2 × 2 × 2 cm) was manually placed in the anterior cingulate cortex (ACC) of the 1-mm isotropic T1- Magnetization Prepared RApid Gradient Echo (MPRAGE) image (repetition time (T_R_) = 1900 ms, echo time (T_E_) = 3.96 ms, flip angle = 8°, slice thickness = 1 mm, 192 slices, field-of-view read = 256 mm) obtained initially [15]. The reproducibility of the voxel placement between visits was assured by taking screenshots of the voxel placed on the ACC in three planes for each participant. The gradient echo shimming was programmed to adjust first- and second-order shims [16]. The semi-Localization by Adiabatic Selective Refocussing (semi-LASER) sequence (T_E_ = 28 ms, T_R_ = 3 s, 128 averages) with variable power radiofrequency pulses with optimized relaxation delay (VAPOR) was used to acquire the spectra [17].

**Fig. 1:**
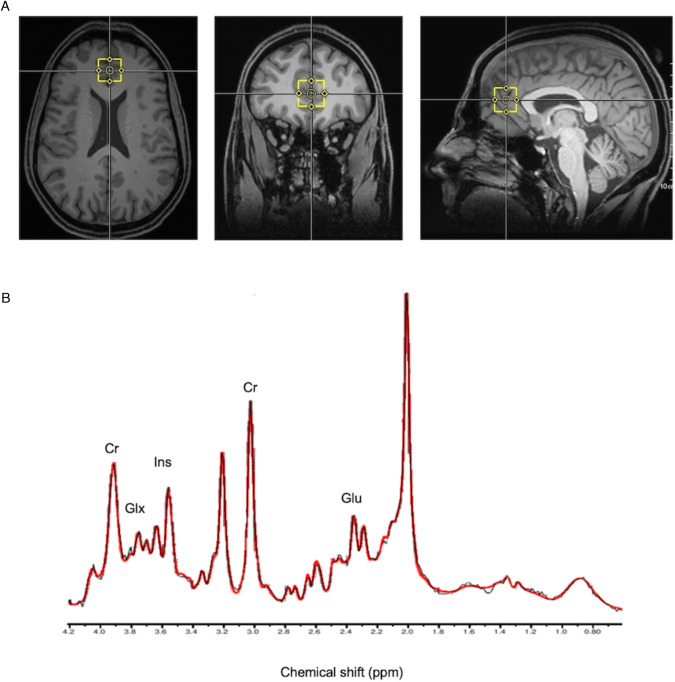
MRS. Voxel placement (**A**) and representative spectra (**B**) from the anterior cingulate cortex (ACC).

The LCModel in MATLAB 2016 was used to quantify inositol (Ins), Glx (glutamate + glutamine), and glutamate (Glu), with total creatine (creatine (Cr) and phosphocreatine (PCr) as reference [18]. Brain extraction was carried out using the Brain Extraction Tool of FMRIB Software Library (FSL) [19]. The cerebrospinal fluid (CSF), white matter (WM), and grey matter (GM) percentages were quantified using FMRIB’s Automated Segmentation Tool (FAST) with MPRAGE images as the input [20]. Then, corrected metabolite concentrations were calculated using the formula: Corrected metabolite concentration = uncorrected metabolite concentration*(GM*43300 + WM*35880 + CSF*55556)/(1−CSF)/55556. Another LCModel output generated was standard deviation (SD) or Cramér-Rao lower bounds, an estimated error of the metabolite measurements. Metabolites with CRLB > 30% were classified as not detected [10]. All paired spectra were visually inspected to detect any significant differences (Fig. 1).

### Depression and anxiety rating scales

Depression and anxiety rating scales were the secondary outcomes of the study. Self-rated questionnaires, a 16-item Self-Report Quick Inventory of Depressive Symptomatology (QIDS-SR16) and a 7-item Generalized Anxiety Disorder (GAD-7) were assessed at baseline and post-treatment. A clinician-rated questionnaire, the Montgomery Asberg Depression Rating Scale (MADRS) was utilised to assess the severity of depression at baseline and at the 7-day visit.

### Selenium levels

Venous blood was obtained from participants on the day 7 visit to assess compliance to treatment. The blood was centrifuged, and the serum was kept in the freezer at −80 °C. After unblinding, the samples of patients taking ebselen were sent to the Department of Laboratory Medicine, University Hospital Southampton NHS Foundation Trust for selenium (a surrogate ebselen measure) analysis. Measurement of total selenium concentrations was conducted by inductively coupled plasma mass spectrometry (ICP-MS) (Perkin Elmer NEXION 300D equipped with a dynamic reaction cell, DRC). The serum was diluted in 0.5% butanol for selenium in a ratio of 1:50. The samples were run against matrix-matched calibration solutions prepared with bovine serum (Sigma-Aldrich). Selenium-78 was measured, and a signal from the analyte isotope was compared against rhodium (internal standard).

### Statistical analysis

All data analyses were performed using IBM SPSS statistics version 29. Comparison between groups was conducted using an independent t-test for continuous variables. For categorical variables, we utilised the chi-square test to assess the differences between groups. Analysis of variance (ANOVA) was conducted for all ETB tasks with treatment (ebselen vs placebo) as a between-subject factor and emotion or valence and time (baseline vs day 7-visit) as two within-subject factors. For FDOT, conditions (unmasked or masked) were an additional within-subject factor. Post hoc independent t-tests between ebselen and placebo (the differences between baseline and treatment visits) were performed if there were any significant interactions. We also performed analysis of co-variance (ANCOVA) with baseline values serving as the covariates. For MRS analysis, we used a two-way ANOVA with time as a within-subject factor and treatment group as a between-subject factor. Significant interactions were followed up with a paired t-test. The differences between visits were used to compare between ebselen and placebo for MADRS, QIDS-SR16 and GAD-7. An additional paired t-test was conducted to explore the changes in each group individually. A Pearson’s product-moment correlation was used to test for correlation between selenium levels and changes in inositol, Glx and glutamate (the differences between baseline and treatment visits) in those receiving ebselen. A *p*-value of <0.05 was considered a significant result. All analyses were conducted blind to treatment allocation.

## Results

Out of 56 participants who completed the baseline assessments, 51 were included in the analysis. Five participants were excluded due to the following factors: identified bipolar depression [1], missing data due to device malfunction [1], possible hypersensitivity to the scanner material [1], COVID-19 infection [1], and non-compliance [1]. No significant differences were observed between treatment groups regarding age, gender, and antidepressant groups (1–2 or >2 previous treatments, including the current episode (Table 1, Table S1). We found no significant difference between ebselen (8/27; 29.6%) and placebo (10/24; 41.7%) groups regarding the correct guess of treatment received (*p* = 0.369 (*χ*^2^)).

**Table 1:** Baseline characteristics and clinical measures of participants included in the study.

Variable	Ebselen (*n* = 27)	Placebo (*n* = 24)
Age	38.3 ± 2.5 (*n* = 27)	37.7 ± 3.3 (*n* = 24)
Gender	Female: 19 (37.3%), male: 8 (15.7%)	Female: 15 (29.4%), male: 9 (17.6%)
Antidepressant groups	1–2: 18 (35.3%), >2: 9 (17.6%)	1–2: 15 (29.4%), >2: 9 (17.6%)
HAM-D (Baseline)	19.6 ± 0.9	19.1 ± 0.8
*Change in clinical measures over 1 week of treatment*		
MADRS	−3.52 ± 1.21	−3.87 ± 1.68
QIDS-SR16	−3.74 ± 1.20	−5.04 ± 1.11
GAD-7	−0.96 ± 0.85	−3.12 + 0.74

Continuous variables are presented in mean ± standard error of mean (SEM) and categorical variables are presented in count (percentage).

### Emotional testing battery

**Fig. 2:**
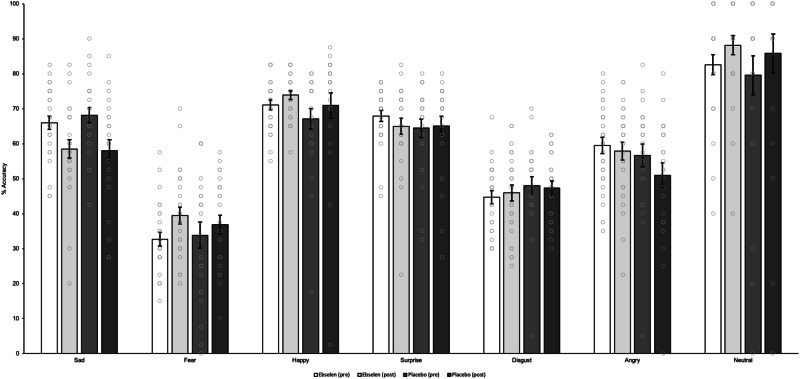
Mean (± SEM) per cent accuracy in the Facial Rcognition Task (FERT). There are no significant effects of treatment (ebselen versus placebo).

There was no three-way interaction between treatment, time and emotion for the accuracy of recognition of facial expressions (F_3.9,192.9_ = 0.73, *p* = 0.57, partial η^2^ = 0.015). There was a significant two-way interaction between time and emotion for the accuracy of recognition of facial expressions (F_3.9,192.9_ = 9.82, *p* < 0.001, partial η^2^ = 0.167), with both groups showing a decrease in accuracy in the detection of sad faces at the day-7 visit. Post hoc independent t-tests showed no significant differences between groups (Fig. 2, Table S2). There were no significant three- (F_2.5,119.8_ = 1.15, *p* = 0.33, partial η^2^ = 0.023) and two-way treatment interactions on the FERT reaction time (Table S3). There was a significant main effect of time (F_1_ = 35.42, *p* < 0.001, partial η^2^ = 0.420), with a decrease in reaction time for all emotions in both groups on the day-7 visit, likely indicating practice effects on the task. There was no three-way interaction between treatment, time and emotion for FERT misclassification (F_3.0,145.0_ = 0.95, *p* = 0.42, partial η^2^ = 0.019). A significant two-way interaction (F_3.0,145.0_ = 10.44, *p* < 0.001, partial η^2^ = 0.176) between time and emotion was observed in the FERT misclassification, where both treatment groups had trends towards a decrease in sad faces misclassification, but an increase in disgust misclassification. However, post hoc independent t-tests showed no significant differences between groups (Table S2).

There was no three-way interaction between treatment, time and emotion for d’ (F_3.3,157.3_ = 0.61, *p* = 0.63, partial η^2^ = 0.013). There was a significant two-way interaction between time and emotion for d’ in the FERT task (F_3.3,157.3_ = 6.05, *p* < 0.001, partial η^2^ = 0.114), with both groups showing a decrease in d’ in detecting sad, disgust and angry faces at the day-7 visit. However, post hoc independent t-tests showed no significant differences between groups (Table S2). There was no three-way interaction between treatment, time and emotion for beta (F_2.0,98.2_ = 0.50, *p* = 0.61, partial η^2^ = 0.01). There was a significant two-way interaction between time and emotion for d’ in the FERT task (F_2.0,98.2_ = 8.50, *p* < 0.001, partial η^2^ = 0.15), with both groups showing an increase in beta values in sad at the day-7 visit, indicating fewer false alarms. However, post hoc independent t-tests showed no significant differences between groups (Table S2). Interestingly, we found a significant difference in post-treatment beta values for fear in ANCOVA (F_1,47_ = 4.64, *p* = 0.04, partial η^2^ = 0.09). A post hoc pairwise analysis showed that the ebselen group (adjusted mean ± SEM = 0.73 ± 0.02) had significantly a lower beta value for fear than placebo (0.80 ± 0.02) with a mean difference of −0.71 (±0.33 SEM, *p* = 0.04) (Table S4). This suggests a bias towards fearful responding.

There was no three-way interaction between treatment, time and emotion for the EREC accuracy (F_1,46_ = 1.00, *p* = 0.32, partial η^2^ = 0.021). A significant two-way interaction between time and emotion was observed in the accuracy of recalling positive and negative self-reference words (F_1,46_ = 7.97, *p* = 0.007, partial η^2^ = 0.148) in the EREC task. Both groups showed trends towards an increase in positive word recall but a decrease in negative word recall on the day-7 visit. However, post hoc independent t-tests showed no significant differences between groups (Table S5).

There was no significant three-way interaction between treatment, time and emotion for the EMEM d’ (F_1,49_ = 0.30, *p* = 0.59, partial η^2^ = 0.01). A significant two-way interaction between time and emotion was observed in d’ (F_1,49_ = 4.47, *p* = 0.04, partial η^2^ = 0.08) in the EMEM task. Both groups showed trends towards an increase in d’ for positive word memory but a decrease in d’ for negative word memory on the day-7 visit. However, post hoc independent t-tests showed no significant differences between groups (Table S6).

There was a significant four-way interaction between time, valence, condition and treatment for the FDOT attentional vigilance score (F_1,49_ = 4.47, *p* = 0.04, partial η^2^ = 0.084). However, there were no significant three-way and two-way interactions between all possible combinations of between- and within-subject factors (Refer to Table S7 for all the values).

No significant three- and two-way interactions were observed in the ECAT (accuracy, reaction time (Table S8)) and EMEM (accuracy, reaction time, misclassification, and beta) tasks (Table S9). ANCOVA for all ETB parameters except beta for the FERT tasks did not show any significant effects of ebselen.

### Magnetic resonance spectroscopy

**Fig. 3:**
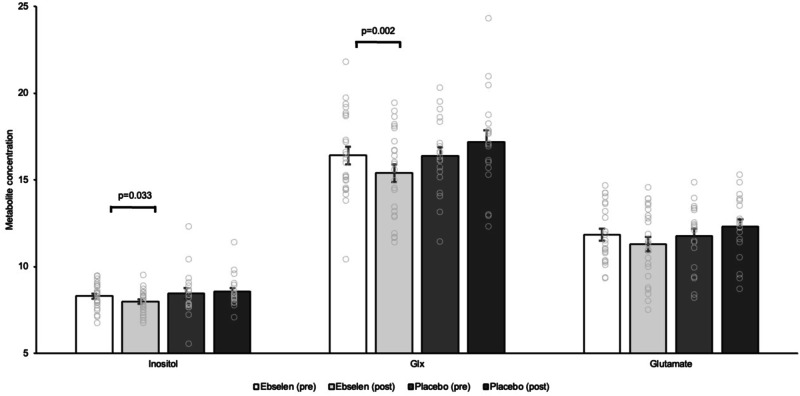
Mean (± SEM) relative concentrations of inositol, glx (glutamate and glutamine combined) and glutamate referenced to total creatine and corrected for cerebrospinal fluid and white and grey matter.

Five out of 48 completed pre-post MRS scan pairs were eliminated due to significant deviations from the expected spectra in one or both visits. The average signal-to-ratio (SNR) for the first and second visits were 43.4 ± 1.4 (mean ± SEM) and 43.12 ± 1.9, respectively. The linewidth for the first and second visits were 8.71 ± 0.30 and 8.79 + 0.33, respectively. The FWHM for the first and second visits were 0.05 ± 0.003 and 0.05 ± 0.002. There was a significant two-way interaction between time and treatment (F_1,41_ = 4.32, *p* = 0.044, partial η^2^ = 0.095) for inositol. A post hoc paired *t*-test analysis revealed a significant decrease in ebselen (*t*(23) = −2.274, *p* = 0.033), but not in placebo-treated participants (*t*(18) = 0.766, *p* = 0.454) (Fig. 3). Glx (a composite measure of glutamate and its precursor and metabolite, glutamine) also showed a significant two-way interaction between time and treatment (F_1,41_ = 6.79, *p* = 0.013, partial η^2^ = 0.142). A post hoc paired *t*-test analysis revealed a significant decrease in participants receiving ebselen (*t*(23) = −3.591, *p* = 0.002), but not in those taking placebo (*t*(18) = 1.144, *p* = 0.268) (Fig. 3). A significant two-way interaction between time and treatment was observed for glutamate (F_1,41_ = 5.75, *p* = 0.021, partial η^2^ = 0.123), but a post hoc paired *t*-test analysis only showed a trend towards significance in the ebselen group (*t*(23) = −1.982, *p* = 0.060). No significant effect of time or treatment was observed on concentrations of the total creatine reference.

### Clinical rating scales

No significant differences in MADRS score changes (*t*(49) = 0.175, *p* = 0.86) were observed between ebselen and placebo over the 7 days of treatment (Table 1). Similarly, we found no significant differences in QIDS-SR16 score changes (*t*(49) = 0.789, *p* = 0.43) between ebselen and placebo. There was a trend in the GAD-7 scores for the ebselen group to show a smaller decrease in GAD-7 score than the placebo group (*t(*49) = 1.889, *p* = 0.07, Table 1). Thus, while the placebo group demonstrated a significant fall in GAD-7 score over the seven days of treatment (*t*(23)= −4.207, *p* < 0.001) this was not the case in the ebselen group (*t*(26) = −1.129, *p* = 0.269).

### Selenium levels and correlation with metabolite concentrations

Selenium levels were measured at 7 days in those who received ebselen treatment. We were not able to obtain blood samples from three participants due to blood withdrawal difficulty and two participants did not wish to give a blood sample. Selenium was present in the blood samples of all remaining 22 participants with a mean value of 9.07 ± 0.72 µmol/L (normal values: 2.99–3.99 µmol/L [21]), indicating the participants’ compliance. The correlation analysis for selenium levels and metabolite concentrations included only 19 participants, as three more participants were excluded due to significant spectra deviations. There were trends towards significant negative correlations between selenium levels and decrease in inositol (*r*(17) = −0.38, *p* = 0.11), but no significant correlations were found between selenium levels and changes in Glx and glutamate concentrations.

### Adverse effects

**Fig. 4:**
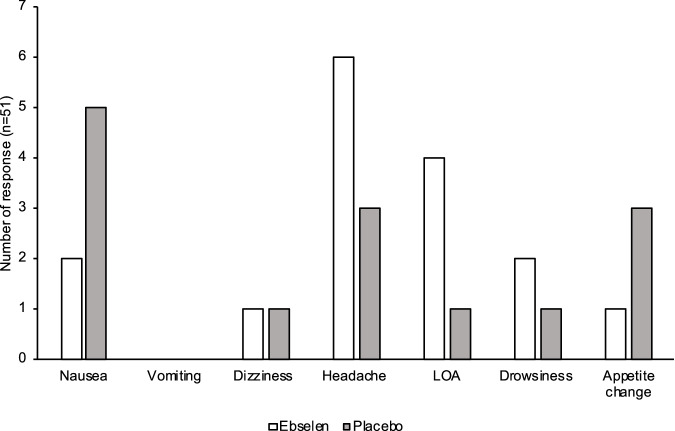
Frequency of adverse effects reported by the participants following ebselen or placebo treatment.

Ebselen was well-tolerated. No serious adverse effects of treatment-related dropouts were observed. Six participants receiving ebselen reported headaches compared to three in those taking placebo (*p* = 0.34). Four participants on ebselen, and one in the placebo group, reported loss of alertness (*p* = 0.20). Otherwise, other side effects were numerically comparable (Fig. 4).

## Discussion

In this study, we were able to confirm a key neurochemical effect of ebselen that we previously described, namely a decrease in inositol concentration in the ACC, measured by MRS. This suggests that the dose of ebselen we employed (600 mg twice daily) produced target engagement, that is, inhibition of IMPase, in this population. However, we found no effect of ebselen on our primary outcome measure, the recognition of emotional faces, as well as other emotional processing parameters in secondary analyses, except for beta values for fear emotion in the FERT task. Beta is a measure of response bias with higher values indicating a more conservative response style. Our study found that ebselen had significantly lower beta values for fear emotion than placebo (moderate effect size), indicating a greater response towards fear in the absence of actual stimulus (more false alarms).

Acute and subacute administration of conventional antidepressant medication such as SSRIs produces positive biases in the recognition of emotional faces in both healthy volunteers and depressed patients. This early action has been linked to subsequent clinical improvement and may be a useful screen for novel antidepressants [14, 22]. Our previous studies in healthy participants indicated that ebselen increased the accuracy of recognising positive facial expressions [12, 13]. However, this was not replicated in the present investigation in depressed patients.

A possible explanation for this lack of effect is that because the depressed patients in the present study were taking antidepressant medication, they could have already experienced a positive shift in emotional processing, even though this had not been associated with significant clinical benefit. In this situation, it may be that there was no scope for further positive biasing in emotional facial recognition by ebselen. It is also possible that in depressed patients, as opposed to healthy controls, ebselen does not produce positive bias in the recognition of facial expressions. A study of ebselen in unmedicated depressed patients will be needed to address this possibility.

The MRS measures also confirmed a previous study in which we found a decrease in Glx in the ACC in healthy participants following subacute ebselen treatment. This is probably attributable to the ability of ebselen to block glutaminase, the enzyme responsible for the synthesis of glutamate. How this might affect ebselen’s potential as an antidepressant augmentation treatment is unclear.

We did not expect to see changes in clinical rating scales over this short period of placebo-controlled treatment though, in fact, some clinical studies have shown that relative to placebo, lithium addition to antidepressants can result in decreases in observer-rated depressive symptomatology over the first week of augmentation therapy [23]. In the present study, there was no difference in change in depression ratings on the MADRS scales between ebselen and placebo between baseline and 7 days. However, there was a trend of ebselen to diminish anxiety scores on the GAD-7 less than placebo. The findings are in parallel with the beta values for fear in the FERT task, suggesting a bias towards fearful responding, and raising the possibility of a mild anxiogenic effect of ebselen after seven days of treatment.

We measured plasma selenium levels as a surrogate ebselen measure, as a previous study confirmed a high correlation between ebselen levels and selenium concentrations [24]. No significant correlations were found between selenium levels and change in MRS metabolite concentration. The result was similar to our previous study in healthy volunteers, where no significant correlation was observed between selenium and inositol levels during ebselen treatment [13]. The differences between plasma and brain levels of ebselen might partly explain this lack of correlation. The measurement of selenium or ebselen concentration in the brain is not possible in humans, so the results from animal studies can provide some insights into the differences. A rodent model study reported that only one-fifth of ebselen accumulated in the brain after a bolus intravenous infusion of ebselen [25].

We found that the current regime of ebselen administration was well-tolerated with no serious adverse events and a side-effect profile similar to placebo. This is similar to findings with ebselen in other studies including one in bipolar patients with mania or hypomania where ebselen was added to ongoing antipsychotic drug treatment [26].

Several limitations in the current study should be addressed in future studies. The evidence regarding the normalization of emotional processing after antidepressant treatment in patients unresponsive to medication is still limited, and more data are required to assess the extent of the reversal of negative biases in this population. The experimental design of our study did not allow us to measure the clinical effectiveness of ebselen, as a longer study period is required to observe changes in depressive symptoms measured using clinical scales. We chose to study ebselen in patients insufficiently helped by conventional antidepressant because this is an accepted use of lithium rather than as a first line antidepressant treatment. However, given the ebselen effects on emotional processing and brain neurochemistry in healthy volunteers, it is possible that unmedicated depressed patients might benefit from ebselen monotherapy.

There could also be a longer-term preventative role for ebselen in that a cohort study in the Finnish population reported that lithium monotherapy reduced hospital readmission in patients with unipolar depression more than conventional antidepressant treatments [27]. However, we should note that while short-term treatment with ebselen is well-tolerated, the longest treatment duration with ebselen in published studies is four weeks [7, 28] and many important side effects related to lithium (thyroid and kidney disease) may develop later.

Interestingly, there may be a role for inositol treatment in preventing some of the adverse effects of lithium [29]. This is because oral inositol administration has little brain penetration and therefore may address the peripheral consequences of inositol depletion without attenuating the CNS actions of lithium. The same would presumably apply to ebselen.

Our experimental medicine study did not find any positive shift in emotional bias in depressed patients with an inadequate response to antidepressant medication. As a positive shift in emotional processing bias was used as a surrogate marker of antidepressant potential in the study, our study did not support any antidepressant potential in depressed patients who were currently on their antidepressants. We previously found that this regime of ebselen administration, given for three weeks, provided evidence of a therapeutic benefit in patients with mania/hypomania [26]. We therefore believe it worthwhile further exploring the role for ebselen in the treatment of bipolar disorder, where drugs with the effectiveness of lithium but with an improved safety profile are needed.

### Supplementary information

Supplemental material

## Data Availability

Data is available on request from the corresponding author.
