# Supplementary material for: Effects of ebselen addition on emotional processing and brain neurochemistry in depressed patients unresponsive to antidepressant medication
Source: Transl Psychiatry. 2024 May 7;14:200. doi: 10.1038/s41398-024-02899-8 (PMC11076504; doi:10.1038/s41398-024-02899-8)
Supplement: Supplementary file 1 — Supplemental material [file 41398_2024_2899_MOESM1_ESM.docx]

Supplementary

Current antidepressant use

Table S1. Percentage and count of current antidepressant use by the participants (n=51)

| Antidepressants | Percentage (count) |
| --- | --- |
| Selective serotonin reuptake inhibitor  Sertraline  Citalopram  Fluoxetine  Escitalopram | 39.2 (20)  21.6 (11)  9.8 (5)  5.9 (3) |
| Serotonin and norepinephrine reuptake inhibitor  Venlafaxine | 7.8 (4) |
| Norepinephrine-dopamine reuptake inhibitor  Bupropion | 2.0 (1) |
| Atypical  Mirtazapine  Agomelatine | 13.7 (7)  3.9 (2) |
| Serotonin modulators  vortioxetine | 2.0 (1) |
| Tricyclic antidepressants  Imipramine | 2.0 (1) |

Emotional Testing Battery

1. FERT

Table S2. Independent t-test for the FERT accuracy, misclassification, reaction times, d prime and beta (n=51).

| Accuracy (percentage) | | | | | | | |
| --- | --- | --- | --- | --- | --- | --- | --- |
| Valence | t | df | p-value | Mean difference | SEM | 95% confidence interval (lower & upper) | |
| Sad | 0.75 | 49 | 0.46 | 2.60 | 3.46 | -4.34 | 9.55 |
| Fear | 1.17 | 49 | 0.25 | 3.74 | 3.21 | -2.70 | 10.18 |
| Happy | -0.44 | 49 | 0.66 | -1.08 | 2.46 | -6.02 | 3.87 |
| Surprise | -1.30 | 49 | 0.20 | -3.59 | 2.76 | -9.14 | 1.96 |
| Disgust | 0.78 | 49 | 0.44 | 1.93 | 2.47 | -3.04 | 6.90 |
| Anger | 1.31 | 49 | 0.20 | 4.06 | 3.10 | -2.17 | 10.30 |
| Neutral | -0.13 | 49 | 0.90 | -0.69 | 5.29 | -11.33 | 9.94 |
| Misclassification (percentage) | | | | | | | |
| Sad | 0.15 | 49 | 0.89 | 0.16 | 1.09 | -2.03 | 2.35 |
| Fear | 1.63 | 49 | 0.11 | 1.05 | 0.65 | -0.25 | 2.35 |
| Happy | 0.80 | 49 | 0.43 | 0.51 | 0.64 | -0.77 | 1.78 |
| Surprise | -0.85 | 49 | 0.40 | -0.56 | 0.67 | -1.90 | 0.78 |
| Disgust | -0.38 | 49 | 0.71 | -0.32 | 0.83 | -1.99 | 1.36 |
| Anger | -0.25 | 49 | 0.81 | -0.15 | 0.63 | -1.41 | 1.11 |
| Neutral | -1.29 | 49 | 0.20 | -1.85 | 1.43 | -4.73 | 1.03 |
| Reaction time (seconds) | | | | | | | |
| Sad | -0.18 | 49 | 0.86 | -0.02 | 0.09 | -0.21 | 0.17 |
| Fear | -0.13 | 49 | 0.90 | -0.02 | 0.15 | -0.31 | 0.28 |
| Happy | 1.62 | 49 | 0.11 | 0.14 | 0.08 | -0.03 | 0.31 |
| Surprise | -0.66 | 49 | 0.52 | -0.20 | 0.30 | -0.81 | 0.41 |
| Disgust | -0.34 | 49 | 0.74 | -0.03 | 0.10 | -0.24 | 0.17 |
| Angry | -1.26 | 49 | 0.21 | -0.14 | 0.11 | -0.37 | 0.08 |
| Neutral | 1.55 | 49 | 0.13 | 0.24 | 0.15 | -0.07 | 0.54 |
| d’ | | | | | | | |
| Sad | 0.54 | 49 | 0.59 | 0.01 | 0.01 | -0.01 | 0.03 |
| Fear | -0.85 | 48 | 0.40 | -0.03 | 0.03 | -0.09 | 0.04 |
| Happy | -0.97 | 49 | 0.34 | -0.01 | 0.01 | -0.03 | 0.01 |
| Surprise | -0.80 | 49 | 0.43 | -0.01 | 0.01 | -0.02 | 0.01 |
| Disgust | 0.21 | 49 | 0.83 | 0.00 | 0.01 | -0.02 | 0.03 |
| Anger | 1.25 | 48 | 0.22 | 0.01 | 0.01 | -0.01 | 0.03 |
| Neutral | 0.33 | 48 | 0.74 | 0.01 | 0.02 | -0.03 | 0.04 |
| Beta | | | | | | | |
| Sad | -0.46 | 49 | 0.65 | -0.03 | 0.06 | -0.14 | 0.09 |
| Fear | -2.03 | 48 | 0.05 | -0.09 | 0.04 | -0.18 | 0.00 |
| Happy | 0.48 | 49 | 0.63 | 0.01 | 0.02 | -0.04 | 0.06 |
| Surprise | 0.91 | 49 | 0.37 | 0.03 | 0.04 | -0.04 | 0.11 |
| Disgust | -0.68 | 49 | 0.50 | -0.03 | 0.05 | -0.13 | 0.06 |
| Anger | 0.86 | 49 | 0.39 | 0.06 | 0.07 | -0.08 | 0.19 |
| Neutral | 0.51 | 49 | 0.61 | 0.08 | 0.15 | -0.23 | 0.38 |

Table S3. Three-way and two-way mixed ANOVA for FERT reaction time (seconds) (n=51).

| Interaction | df | F | Sig | Partial Eta squared |
| --- | --- | --- | --- | --- |
| Three-way interactions | | | | |
| Time * Valence * Treatment Group | 2.445 | 1.148 | 0.328 | 0.023 |
| Error (Time * Valence) | 119.8 |  |  |  |
| Two-way interactions | | | | |
| Time * Treatment Group | 1 | 0.004 | 0.952 | 0.000 |
| Error (Time) | 49 |  |  |  |
| Valence * Treatment Group | 3.063 | 0.285 | 0.840 | 0.006 |
| Error (Valence) | 150.1 |  |  |  |
| Time * Valence | 2.445 | 4.513 | 0.008* | 0.084 |
| Error (Time * Valence) | 119.8 |  |  |  |

Table S4. Analysis of Covariance for FERT, ECAT, EREC, FDOT, and EMEM.

| Valence | ANCOVA statistics | Unadjusted | | Adjusted | |
| --- | --- | --- | --- | --- | --- |
|  |  | Ebselen (mean + SD) | Placebo (mean + SD) | Ebselen (mean + SE) | Placebo (mean + SE) |
| FERT | | | | | |
| Accuracy (%) | | | | | |
| Sad | F(1,48)=0.36, p=0.55, partial η^2^=0.01 | 58.52 + 13.56 | 58.02 + 15.07 | 59.25 + 2.34 | 57.20 + 2.48 |
| Fear | F(1,48)=1.40, p=0.24, partial η^2^=0.03 | 39.44 + 12.64 | 36.88 + 13.17 | 39.77 + 1.88 | 36.51 + 2.00 |
| Happy | F(1,48)=0.06, p=0.81, partial η^2^=0.001 | 73.89 + 6.63 | 70.94 + 17.67 | 72.22 + 1.70 | 72.82 + 1.80 |
| Surprise | F(1,48)=1.06, p=0.31, partial η^2^=0.02 | 65.00 + 12.03 | 65.10 + 13.36 | 63.71 + 1.88 | 66.55 + 1.99 |
| Disgust | F(1,48)=0.13, p=0.72, partial η^2^=0.003 | 45.93 + 11.79 | 47.29 + 9.78 | 46.95 + 1.55 | 46.14 + 1.65 |
| Anger | F(1,48)=2.32, p=0.13, partial η^2^=0.05 | 57.87 + 13.33 | 50.94 + 17.57 | 56.79 + 2.08 | 52.15 + 2.21 |
| Neutral | F(1,48)=0.01, p=0.92, partial η^2^=0.00 | 88.15 + 14.15 | 85.83 + 27.17 | 87.30 + 3.26 | 86.79 + 3.46 |
| Reaction time (s) | | | | | |
| Sad | F(1,48)=0.03, p=0.86, partial η^2^=0.001 | 1.58 + 0.41 | 1.58 + 0.38 | 1.57 + 0.05 | 1.59 + 0.05 |
| Fear |  | 1.82 + 0.36 | 1.86 + 0.53 |  |  |
| Happy | F(1,48)=2.45, p=0.12, partial η^2^=0.05 | 1.63 + 0.40 | 1.57 + 0.46 | 1.68 + 0.05 | 1.54 + 0.05 |
| Surprise |  | 1.63 + 0.39 | 1.64 + 0.50 |  |  |
| Disgust | F(1,48)=0.02, p=0.89, partial η^2^=0.000 | 1.72 + 0.36 | 1.69 + 0.52 | 1.70 + 0.06 | 1.71 + 0.07 |
| Anger |  | 1.74 + 0.39 | 1.74 + 0.55 |  |  |
| Neutral |  | 1.44 + 0.54 | 1.29 + 0.44 |  |  |
| Misclassification (%) | | | | | |
| Sad | F(1,48)=0.01, p=0.94, partial η^2^=0.00 | 6.23 + 4.70 | 6.45 + 6.75 | 6.37 + 0.71 | 6.29 + 0.76 |
| Fear | F(1,48)=2.64, p=0.11, partial η^2^=0.05 | 3.92 + 3.39 | 2.88 + 2.82 | 3.92 + 0.44 | 2.87 + 0.47 |
| Happy |  | 0.19 + 0.46 | 1.23 + 4.53 |  |  |
| Surprise | F(1,48)=0.25, p=0.62, partial η^2^=0.01 | 5.22 + 2.22 | 5.18 + 2.88 | 5.06 + 0.42 | 5.36 + 0.45 |
| Disgust | F(1,48)=3.61, p=0.06, partial η^2^=0.07 | 3.63 + 1.69 | 5.71 + 3.47 | 3.91 + 0.52 | 5.40 + 0.55 |
| Anger |  | 3.32 + 1.97 | 3.55 + 3.12 |  |  |
| Neutral | F(1,48)=1.11, p=0.30, partial η^2^=0.02 | 24.03 + 6.77 | 23.85 + 8.41 | 23.24 + 0.97 | 24.74 + 1.03 |
| d’ | | | | | |
| Sad | F(1,48)=0.25, p=0.62, partial η^2^=0.01 | 0.86 + 0.04 | 0.86 + 0.05 | 0.87 + 0.01 | 0.86 + 0.01 |
| Fear |  | 0.82 + 0.05 | 0.81 + 0.07 |  |  |
| Happy | F(1,48)=0.004, p=0.95, partial η^2^=0.000 | 0.93 + 0.02 | 0.92 + 0.05 | 0.93 + 0.01 | 0.93 + 0.01 |
| Surprise |  | 0.89 + 0.03 | 0.89 + 0.05 |  |  |
| Disgust |  | 0.84 + 0.03 | 0.83 + 0.04 |  |  |
| Anger | F(1,47)=2.11, p=0.15, partial η^2^=0.04 | 0.88 + 0.04 | 0.86 + 0.04 | 0.88 + 0.01 | 0.86 + 0.01 |
| Neutral | F(1,47)=0.01, p=0.94, partial η^2^=0.000 | 0.90 + 0.04 | 0.90 + 0.07 | 0.90 + 0.01 | 0.90 + 0.01 |
| Beta | | | | | |
| Sad | F(1,48)=0.19, p=0.67, partial η^2^=0.004 | 0.62 + 0.24 | 0.63 + 0.26 | 0.61 + 0.04 | 0.64 + 0.04 |
| Fear | F(1,47)=4.64, p=0.04*, partial η^2^=0.09 | 0.74 + 0.16* | 0.78 + 0.22* | 0.73 + 0.02* | 0.80 + 0.02* |
| Happy |  | 0.98 + 0.04 | 0.93 + 0.17 |  |  |
| Surprise | F(1,48)=0.19, p=0.66, partial η^2^=0.004 | 0.63 + 0.14 | 0.64 + 0.15 | 0.64 + 0.02 | 0.62 + 0.03 |
| Disgust | F(1,48)=2.39, p=0.13, partial η^2^=0.05 | 0.75 + 0.10 | 0.66 + 0.16 | 0.73 + 0.03 | 0.67 + 0.03 |
| Anger |  | 0.76 + 0.13 | 0.68 + 0.40 |  |  |
| Neutral | F(1,48)=1.64, p=0.21, partial η^2^=0.03 | -0.45 + 0.49 | -0.67 + 0.51 | -0.47 + 0.09 | - 0.64 + 0.10 |
| ECAT | | | | | |
| Accuracy (%) | | | | | |
| Positive | F(1,48)=1.33, p=0.25, partial η^2^=0.03 | 95.37 + 8.43 | 94.17 + 6.37 | 95.72 + 1.16 | 93.77 + 1.23 |
| Negative | F(1,48)=0.00, p=0.99, partial η^2^=0.000 | 97.78 + 4.67 | 95.42 + 11.97 | 96.68 + 1.19 | 96.65 + 1.27 |
| Reaction time (seconds) | | | | | |
| Positive | F(1,48)=0.09, p=0.77, partial η^2^=0.002 | 0.99 + 0.26 | 1.01 + 0.25 | 1.01 + 0.04 | 0.99 + 0.04 |
| Negative |  | 1.02 + 0.21 | 1.05 + 0.23 |  |  |
| EREC | | | | | |
| Correct recall (count) | | | | | |
| Positive | F(1,45)=1.40, p=0.24, partial η^2^=0.03 | 5.58 + 2.50 | 4.86 + 1.83 | 5.57 + 0.40 | 4.88 + 0.43 |
| Negative | F(1,45)=0.09, p=0.76, partial η^2^=0.02 | 3.31 + 2.13 | 2.82 + 1.76 | 3.16 + 0.36 | 3.00 + 0.39 |
| Incorrect recall (count) | | | | | |
| Positive | F(1,45)=1.43, p=0.24, partial η^2^=0.03 | 2.77 + 1.86 | 2.05 + 1.84 | 2.68 + 0.30 | 2.15 + 0.33 |
| Negative | F(1,45)=3.04, p=0.09, partial η^2^=0.06 | 1.96 + 2.38 | 1.05 + 1.33 | 1.98 + 0.37 | 1.03 + 0.40 |
| FDOT (attentional vigilance score) | | | | | |
| Happy | | | | | |
| Unmasked | F(1,48)=0.002, p=0.96, partial η^2^=0.000 | -0.03 + 0.04 | -0.03 + 0.06 | -0.03 + 0.01 | -0.03 + 0.01 |
| Masked | F(1,48)=2.63, p=0.11, partial η^2^=0.05 | -0.003 + 0.04 | -0.003 + 0.05 | -0.004 + 0.01 | -0.02 + 0.01 |
| Fear |  |  |  |  |  |
| Unmasked | F(1,48)=0.83, p=0.37, partial η^2^=0.02 | 0.01 + 0.06 | 0.03 + 0.05 | 0.01 + 0.01 | 0.03 + 0.01 |
| Masked | F(1,48)=5.82, p=0.02, partial η^2^=0.11 | 0.03 + 0.06 | -0.007 + 0.04 | 0.03 + 0.01 | -0.01 + 0.01 |
| EMEM | | | | | |
| Accuracy (%) | | | | | |
| Positive | F(1,48)=0.32, p=0.58, partial η^2^=0.01 | 78.32 + 10.85 | 78.35 + 9.76 | 79.05 + 1.84 | 77.53 + 1.95 |
| Negative | F(1,48)=0.002, p=0.96, partial η^2^=0.000 | 76.06 + 11.85 | 78.15 + 11.94 | 76.97 + 2.19 | 77.12 + 2.33 |
| Reaction time (seconds) | | | | | |
| Positive |  | 1.11 + 0.22 | 1.14 + 0.21 |  |  |
| Negative | F(1,48)=0.01, p=0.94, partial η^2^=0.000 | 1.12 + 0.21 | 1.18 + 0.22 | 1.15 + 0.03 | 1.15 + 0.03 |
| Misclassification (%) | | | | | |
| Positive | F(1,48)=0.32, p=0.58, partial η^2^=0.01 | 21.68 + 10.85 | 21.65 + 9.76 | 20.95 + 1.84 | 22.47 + 1.95 |
| Negative | F(1,48)=0.002, p=0.96, partial η^2^=0.000 | 23.94 + 11.85 | 21.85 + 11.94 | 23.03 + 2.19 | 22.88 + 2.33 |
| d’ | | | | | |
| Positive | F(1,48)=0.10, p=0.75, partial η^2^=0.002 | 0.84 + 0.09 | 0.86 + 0.05 | 0.85 + 0.01 | 0.86 + 0.02 |
| Negative | F(1,48)=0.05, p=0.82, partial η^2^=0.001 | 0.88 + 0.07 | 0.91 + 0.07 | 0.89 + 0.01 | 0.90 + 0.01 |
| Beta | | | | | |
| Positive | F(1,48)=0.004, p=0.95, partial η^2^=0.000 | 0.07 + 0.43 | 0.06 + 0.34 | 0.07 + 0.07 | 0.06 + 0.08 |
| Negative | F(1,48)=0.39, p=0.53, partial η^2^=0.01 | 0.32 + 0.37 | 0.42 + 0.42 | 0.34 + 0.07 | 0.40 + 0.08 |

*Indicates a significant value of p<0.05

In case of violation of assumption for regression of coefficient slope, and we only provide the mean and standard error values of the second visit.

1. EREC

Table S5. Independent t-test for the EREC correct and incorrect words recalled accuracy (n=48).

| Valence | t | df | p-value | Mean difference | SEM | 95% confidence interval (lower & upper) | |
| --- | --- | --- | --- | --- | --- | --- | --- |
| Positive correct | 1.03 | 40.6 | 0.31 | 0.66 | 0.65 | -0.64 | 1.97 |
| Negative correct | -0.46 | 46 | 0.65 | -0.28 | 0.61 | -1.51 | 0.95 |
| Positive incorrect | 0.87 | 46 | 0.39 | 0.41 | 0.48 | -0.54 | 1.37 |
| Negative incorrect | 1.63 | 46 | 0.11 | 0.99 | 0.61 | -0.23 | 2.22 |

1. EMEM

Table S6. Independent t-test for the EMEM d prime (n=51).

| Valence | t | df | p-value | Mean difference | SEM | 95% confidence interval (lower & upper) | |
| --- | --- | --- | --- | --- | --- | --- | --- |
| Positive | 0.81 | 49 | 0.42 | 0.02 | 0.03 | -0.03 | 0.07 |
| Negative | 0.17 | 49 | 0.86 | 0.003 | 0.02 | -0.03 | 0.04 |

1. FDOT

Table S7. Three-way mixed ANOVA for attentional vigilance (n=51).

| Interaction | df | F | Sig | Partial Eta squared |
| --- | --- | --- | --- | --- |
| Four-way interaction | | | | |
| Time * Valence * Condition * Treatment Group | 1 | 4.47 | 0.04* | 0.08 |
| Error (Time * Valence * Condition) | 49 |  |  |  |
| Three-way interaction | | | | |
| Time * Valence * Treatment Group | 1 | 0.25 | 0.62 | 0.01 |
| Error (Time * Valence) | 49 |  |  |  |
| Time * Condition * Treatment Group | 1 | 0.73 | 0.40 | 0.02 |
| Error (Time * Condition) | 49 |  |  |  |
| Time * Valence * Treatment Group | 1 | 0.25 | 0.62 | 0.01 |
| Error (Time * Valence) | 49 |  |  |  |
| Valence * Condition * Treatment Group | 1 | 0.61 | 0.44 | 0.01 |
| Error (Valence * Condition) | 49 |  |  |  |
| Two-way interactions | | | | |
| Time * Treatment Group | 1 | 0.59 | 0.45 | 0.01 |
| Error (Time) | 49 |  |  |  |
| Valence * Treatment Group | 1 | 0.02 | 0.90 | 0.00 |
| Error (Valence) | 49 |  |  |  |
| Condition * Treatment Group | 1 | 4.01 | 0.05 | 0.08 |
| Error (Condition) | 49 |  |  |  |
| Time * Valence | 1 | 0.29 | 0.59 | 0.01 |
| Error (Time) | 49 |  |  |  |
| Valence * Condition | 1 | 1.09 | 0.30 | 0.02 |
| Error (Valence) | 49 |  |  |  |
| Time * Condition | 1 | 0.03 | 0.86 | 0.00 |
| Error (Time) | 49 |  |  |  |

1. ECAT (accuracy and reaction time)

Table S8. Three-way mixed ANOVA for ECAT accuracy and reaction time (n=51).

| Accuracy (percentage) | | | | |
| --- | --- | --- | --- | --- |
| Interaction | df | F | Sig | Partial Eta squared |
| Three-way interaction | | | | |
| Time * Valence * Treatment Group | 1 | 1.47 | 0.23 | 0.03 |
| Error (Time * Valence) | 49 |  |  |  |
| Two-way interactions | | | | |
| Time * Treatment Group | 1 | 0.51 | 0.48 | 0.01 |
| Error (Time) | 49 |  |  |  |
| Valence * Treatment Group | 1 | 1.17 | 0.28 | 0.02 |
| Error (Valence) | 49 |  |  |  |
| Time * Valence | 1 | 0.01 | 0.94 | 0.00 |
| Error (Time* Valence) | 49 |  |  |  |
| Reaction time (seconds) | | | | |
| Three-way interaction | | | | |
| Time * Valence * Treatment Group | 1 | 0.02 | 0.89 | 0.0 |
| Error (Time * Valence) | 49 |  |  |  |
| Two-way interactions | | | | |
| Time * Treatment Group | 1 | 0.63 | 0.43 | 0.01 |
| Error (Time) | 49 |  |  |  |
| Valence * Treatment Group | 1 | 0.22 | 0.64 | 0.00 |
| Error (Valence) | 49 |  |  |  |
| Time * Valence | 1 | 0.30 | 0.59 | 0.01 |
| Error (Time* Valence) | 49 |  |  |  |

1. EMEM

Table S9. Three-way mixed ANOVA for EMEM accuracy, reaction time and misclassification (n=51).

| Interaction | df | F | Sig | Partial Eta squared |
| --- | --- | --- | --- | --- |
| Accuracy (percentage) | | | | |
| Three-way interaction | | | | |
| Time * Valence * Treatment Group | 1 | 0.23 | 0.63 | 0.01 |
| Error (Time * Valence) | 49 |  |  |  |
| Two-way interactions | | | | |
| Time * Treatment Group | 1 | 0.78 | 0.38 | 0.02 |
| Error (Time) | 49 |  |  |  |
| Valence * Treatment Group | 1 | 0.48 | 0.49 | 0.01 |
| Error (Valence) | 49 |  |  |  |
| Time * Valence | 1 | 2.51 | 0.12 | 0.05 |
| Error (Time * Valence) | 49 |  |  |  |
| Reaction time (seconds) | | | | |
| Three-way interaction | | | | |
| Time * Valence * Treatment Group | 1 | 0.16 | 0.70 | 0.00 |
| Error (Time * Valence) | 49 |  |  |  |
| Two-way interactions | | | | |
| Time * Treatment Group | 1 | 0.77 | 0.38 | 0.02 |
| Error (Time) | 49 |  |  |  |
| Valence * Treatment Group | 1 | 0.87 | 0.36 | 0.02 |
| Error (Valence) | 49 |  |  |  |
| Time * Valence | 1 | 0.11 | 0.74 | 0.00 |
| Error (Time * Valence) | 49 |  |  |  |
| Misclassifications (percentage) | | | | |
| Three-way interaction | | | | |
| Time * Valence * Treatment Group | 1 | 0.23 | 0.63 | 0.01 |
| Error (Time * Valence) | 49 |  |  |  |
| Two-way interactions | | | | |
| Time * Treatment Group | 1 | 0.78 | 0.38 | 0.02 |
| Error (Time) | 49 |  |  |  |
| Valence * Treatment Group | 1 | 0.48 | 0.49 | 0.01 |
| Error (Valence) | 49 |  |  |  |
| Time * Valance | 1 | 2.51 | 0.12 | 0.05 |
| Error (Time * Valence) | 49 |  |  |  |
| d’ | | | | |
| Three-way interaction | | | | |
| Time * Valence * Treatment Group | 1 | 0.30 | 0.59 | 0.01 |
| Error (Time * Valence) | 49 |  |  |  |
| Two-way interactions | | | | |
| Time * Treatment Group | 1 | 0.78 | 0.38 | 0.02 |
| Error (Time) | 49 |  |  |  |
| Valence * Treatment Group | 1 | 0.11 | 0.74 | 0.00 |
| Error (Valence) | 49 |  |  |  |
| Time * Valance | 1 | 4.47 | 0.04* | 0.08 |
| Error (Time * Valence) | 49 |  |  |  |
| Beta | | | | |
| Three-way interaction | | | | |
| Time * Valence * Treatment Group | 1 | 0.01 | 0.95 | 0.00 |
| Error (Time * Valence) | 49 |  |  |  |
| Two-way interactions | | | | |
| Time * Treatment Group | 1 | 0.001 | 0.97 | 0.00 |
| Error (Time) | 49 |  |  |  |
| Valence * Treatment Group | 1 | 3.11 | 0.08 | 0.06 |
| Error (Valence) | 49 |  |  |  |
| Time * Valance | 1 | 0.10 | 0.75 | 0.00 |
| Error (Time* Valence) | 49 |  |  |  |
